# Incident transient ischemic attack and subsequent cardiovascular events and mortality in patients with newly diagnosed type 2 diabetes: a population-based cohort study

**DOI:** 10.3389/fendo.2026.1938124

**Published:** 2026-10-28

**Authors:** Lingxuan Chen, Zhihai Zhang, Baozhen Lai

**Affiliations:** 1 Department of Traditional Chinese Medicine, The First Affiliated Hospital of Xiamen University, Xiamen, China; 2 Center of Integrated Chinese and Western Medicine, The First Affiliated Hospital of Xiamen University, School of Medicine, Xiamen University, Xiamen, China

**Keywords:** cardiovascular outcomes, Chinese, cohort study, time-varying exposure, transient ischemic attack, type 2 diabetes mellitus

## Abstract

**Background:**

Transient ischemic attack (TIA) is an established warning sign for stroke, but its association with broader cardiovascular outcomes during the course of newly diagnosed type 2 diabetes mellitus (T2DM) remains unclear.

**Methods:**

This population-based cohort included 232,688 adults with newly diagnosed T2DM in the Xiamen Regional Healthcare Database during 2010–2025. Follow-up began at T2DM diagnosis, with hospitalized TIA modeled as a time-varying exposure. The primary outcome was a cardiovascular composite of myocardial infarction (MI), stroke, heart failure (HF), or cardiovascular mortality. Adjusted Cox models estimated hazard ratios (HRs) and 95% confidence intervals (CIs). Analyses examined exploratory subgroup interactions, age-specific absolute rates, exposure delays, smoking complete cases, and models excluding covariates with substantial missingness. Primary discharge and repeated-TIA definitions and ischemic stroke were also assessed.

**Results:**

During follow-up, 11,153 participants experienced hospitalized TIA. Adjusted hazard ratios (95% confidence intervals) were 2.07 (1.96–2.20) for the cardiovascular composite, 1.98 (1.87–2.10) for all-cause mortality, 2.31 (2.09–2.55) for cardiovascular mortality, 1.55 (1.48–1.63) for HF, and 2.46 (2.32–2.61) for stroke. MI was not significantly associated with TIA (1.10; 0.99–1.22). Exploratory interactions involved age and glycated hemoglobin (HbA1c) for the composite, and sex, age, and HbA1c for stroke. Younger participants had larger relative composite and stroke associations, whereas older participants had higher unadjusted absolute event rates. Composite and stroke associations remained elevated across 7–180-day exposure delays; at 180 days, HRs were 1.69 (1.58–1.80) and 1.92 (1.79–2.06), respectively. Associations with the composite, mortality, stroke, and HF persisted across missing-data analyses and alternative TIA definitions. The ischemic stroke HR was 2.45 (2.31–2.60).

**Conclusion:**

In patients with T2DM, hospitalized TIA was associated with higher subsequent hazards of the four-component cardiovascular composite, mortality, stroke, and heart failure in time-varying analyses. These findings support hospitalized TIA as a prognostic marker of broader cardiovascular risk in this population.

## Introduction

Type 2 diabetes mellitus (T2DM) is associated with a substantial burden of atherosclerotic cardiovascular disease and heart failure (HF), contributing to premature mortality (1–4). Despite advances in glycemic management and cardiovascular prevention, cardiovascular morbidity remains a major challenge in this population (1, 2, 4). Characterizing the prognosis associated with vascular events that emerge after diabetes diagnosis is therefore important for understanding the cardiovascular burden throughout the course of T2DM.

Transient ischemic attack (TIA) is a transient episode of neurological dysfunction caused by focal cerebral, spinal cord, or retinal ischemia without acute infarction (5, 6). Although symptoms resolve, TIA is an established warning sign for stroke, with particularly high risk during the first days and weeks and persistent risk over longer follow-up (6–11). Its prognostic significance also extends to myocardial infarction (MI), other vascular events, and vascular mortality (10, 12, 13). Diabetes is included in early stroke risk assessment after TIA and is associated with poorer outcomes after TIA or stroke (14, 15). The atherosclerosis, endothelial dysfunction, and prothrombotic abnormalities associated with T2DM affect multiple vascular beds (4, 16–18), providing a rationale for examining cardiovascular outcomes beyond stroke in this population.

However, much of the evidence on TIA prognosis comes from cohorts enrolled at an index TIA or minor stroke (7–13). Other studies have examined diabetes as a prognostic factor among patients presenting with cerebral ischemia (14, 15). These studies provide limited insight into the prognostic associations of TIA arising during follow-up from T2DM diagnosis. In particular, the associations of incident hospitalized TIA with both cerebrovascular and noncerebrovascular outcomes, especially HF, remain incompletely characterized in patients with newly diagnosed T2DM. To address this gap, follow-up should begin at T2DM diagnosis and account for the timing of incident TIA.

We therefore used the Xiamen Regional Healthcare Database to evaluate associations between incident hospitalized TIA and subsequent cardiovascular outcomes and mortality in adults with newly diagnosed T2DM. In this population-based cohort, TIA was modeled as a time-varying exposure using Cox regression. The primary outcome was a four-component cardiovascular composite comprising MI, stroke, HF, or cardiovascular mortality. Secondary outcomes included all-cause mortality and the individual composite components. We also assessed exploratory effect modification, age-specific absolute event rates, and robustness to delayed exposure classification, alternative TIA definitions, and missing-data handling.

## Materials and methods

### Data source and study design

We conducted a retrospective, population-based cohort study using the Xiamen Regional Healthcare Database, which links healthcare records for residents of Xiamen, China (19). The database integrates demographic information, inpatient and outpatient encounters, laboratory results, medication prescriptions, and mortality records. Diagnoses are coded using the International Classification of Diseases, Tenth Revision (ICD-10), and medications use the Anatomical Therapeutic Chemical (ATC) classification. All data were de-identified before analysis.

### Study population

We identified 257,204 patients with newly diagnosed T2DM between January 1, 2010, and September 1, 2025. Newly diagnosed T2DM was operationally defined as the first T2DM diagnosis recorded in the database, and this date was the index date and cohort entry date. Available historical diagnosis and prescription records, including records before 2010, were reviewed for evidence of pre-existing diabetes. A uniform historical observation period could not be established for every participant. Exclusions comprised severe kidney disease or estimated glomerular filtration rate (eGFR) <30 mL/min/1.73 m²; recorded prior MI, stroke, HF, or TIA; and age <18 years. Prior events were assessed using all available pre-index records, including those before 2010. The cohort was restricted to adults without advanced kidney disease to reduce heterogeneity associated with its cardiovascular prognosis and treatment context. After exclusions, 232,688 participants remained, of whom 11,153 had a hospitalized TIA during follow-up and 221,535 did not (**Figure 1**).

**Figure 1:**
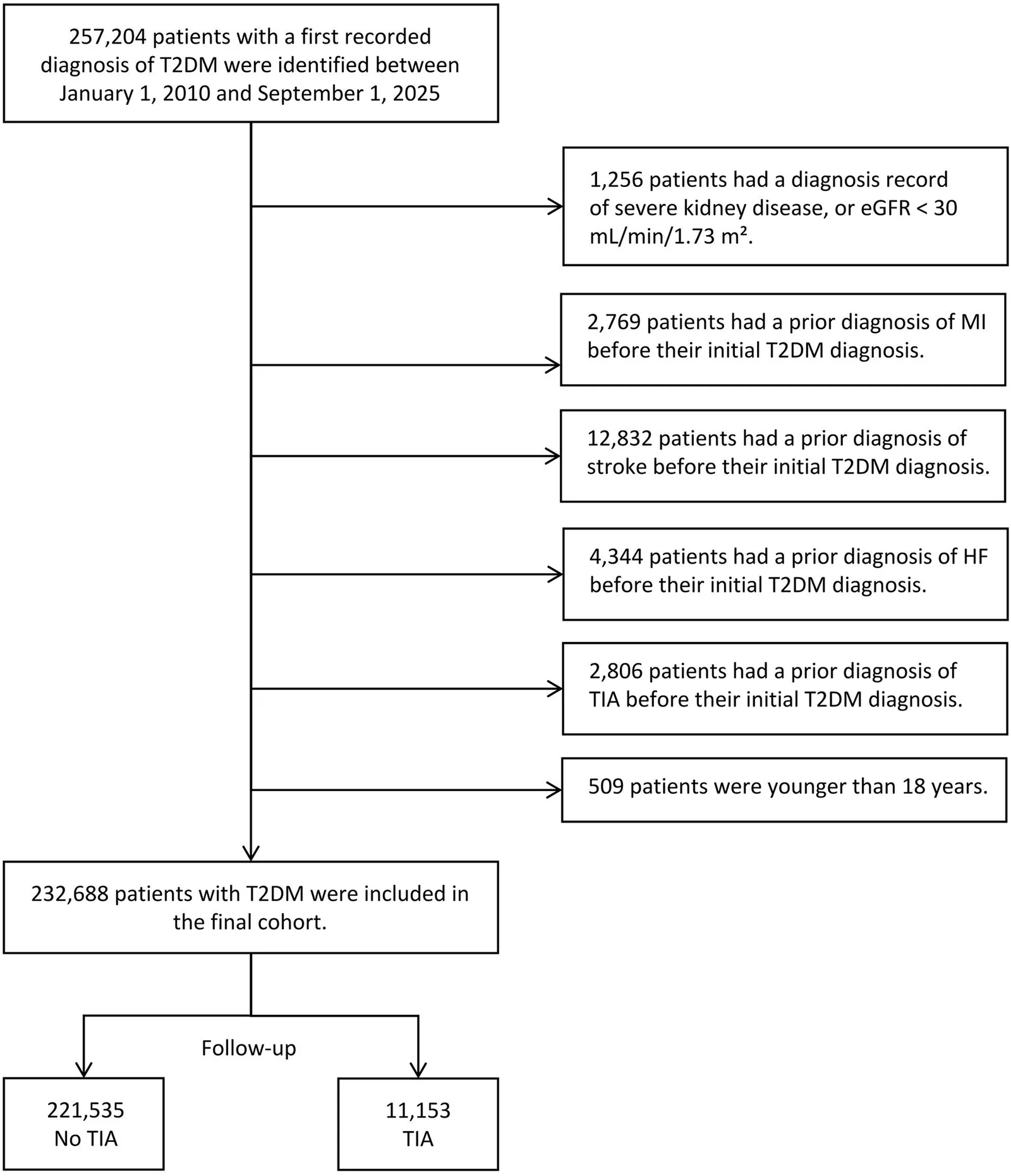
Flowchart of participants included in the study. Abbreviations: eGFR, estimated glomerular filtration rate; HF, heart failure; MI, myocardial infarction; T2DM, type 2 diabetes mellitus; TIA, transient ischemic attack.

### Exposure ascertainment

The exposure was the first recorded hospitalized TIA after cohort entry, identified using ICD-10 code G45. Participants with recorded TIA before cohort entry were excluded. TIA was modeled as a time-varying exposure rather than assigning participants to fixed exposure groups at baseline. Participants contributed unexposed person-time until the first hospitalized TIA and exposed person-time thereafter, subject to outcome-specific follow-up. Events occurring on the same calendar date as TIA were not classified as post-TIA events and remained in the unexposed period. The exposure definition was limited to hospitalized TIA.

### Outcome ascertainment and follow-up

The primary outcome was the first occurrence of a four-component cardiovascular composite comprising MI (ICD-10 I21), stroke (I60–I64), HF (I50), or cardiovascular mortality. Cardiovascular mortality was identified using ICD-10 codes I00–I09, I11, I13, I20–I25, I30–I52, and I60–I69. HF was explicitly included to capture the broader cardiovascular burden relevant to T2DM. Secondary outcomes were all-cause mortality and each component of the composite. An additional analysis evaluated ischemic stroke separately.

All participants entered follow-up at T2DM diagnosis, irrespective of subsequent TIA status. Follow-up was defined separately for each outcome and ended at its first occurrence, death, or September 1, 2025, whichever came first. For all-cause mortality, follow-up ended at death or administrative censoring. For the cardiovascular composite, cardiovascular death counted as an event and non-cardiovascular death ended follow-up without a composite event. For each nonfatal outcome, death before that outcome ended follow-up without an event. Non-cardiovascular death likewise ended follow-up without a cardiovascular mortality event. Participants who experienced an outcome before TIA exited that outcome-specific risk set and did not re-enter after TIA. A preceding event for one outcome did not automatically terminate follow-up for other outcomes.

### Covariates

Baseline covariates were assessed during the year before T2DM diagnosis, using the same window for all participants. Age was defined at cohort entry. Covariates were selected on clinical grounds and included demographic and socioeconomic characteristics, lifestyle factors, medication use, diabetes complications, comorbidities, anthropometric measures, and laboratory indicators. Baseline covariates remained fixed during follow-up; only TIA exposure status was updated over time.

Demographic and socioeconomic covariates included sex, age, elderly status, ethnicity, marital status, and insurance type. Elderly status was defined as age ≥60 years. Lifestyle covariates included smoking history and drinking history. Medication use during the pre-index year was classified as yes or no based on prescription records and ATC codes. Glucose-lowering drugs included insulin, metformin, sulfonylureas, alpha-glucosidase inhibitors (AGIs), thiazolidinediones, glucagon-like peptide-1 receptor agonists (GLP-1 RAs), sodium-glucose cotransporter-2 inhibitors (SGLT-2i), dipeptidyl peptidase-4 inhibitors (DPP-4i), and glinides. Other medications included aspirin, proton pump inhibitors, diuretics, β-blocking agents, calcium channel blockers, angiotensin-converting enzyme inhibitors (ACE inhibitors), angiotensin receptor blockers (ARBs), statins, other lipid-modifying agents, and H2-receptor antagonists.

Diabetes-related complications included diabetic retinopathy, diabetic nephropathy, diabetic neuropathy, and diabetic foot. Other comorbidities included mood disorders, anxiety, psychosis, angina pectoris, atrial fibrillation, hypertension, venous thromboembolism, peripheral vascular disease, chronic kidney disease, hyperlipidemia, liver disease, chronic obstructive pulmonary disease (COPD), asthma, thyroid disease, osteoporosis, osteoarthritis, rheumatoid arthritis, gout, head injury, central nervous system infections, fracture, malignant neoplasms, epilepsy, and Alzheimer’s disease. Comorbidities and diabetes-related complications were identified using relevant ICD-10 diagnosis codes recorded during the baseline period.

Anthropometric and laboratory covariates included body mass index (BMI), glycated hemoglobin (HbA1c), total cholesterol (TC), triglycerides (TG), high-density lipoprotein (HDL), low-density lipoprotein (LDL), creatinine (Crea), and uric acid (UA). Laboratory variables underwent data-quality and plausibility checks before analysis. Values outside prespecified clinically plausible ranges were considered invalid and set to missing. Missing categorical information was retained as an unknown category to preserve participants without documented values. Missing BMI and laboratory values were handled using multiple imputation by chained equations. Missingness before imputation and the handling of each variable are reported in **Supplementary Table 1**.

### Statistical analysis

Baseline characteristics were summarized according to whether participants experienced a hospitalized TIA during follow-up. This classification was used for descriptive purposes only and was not treated as a baseline exposure in the outcome analyses. Continuous variables were presented as medians with interquartile ranges and compared using Wilcoxon rank-sum tests. Categorical variables were presented as counts and percentages and compared using Pearson’s chi-square tests.

The primary analyses used multivariable Cox proportional hazards models in which hospitalized TIA was treated as a time-varying exposure. Follow-up began at the date of T2DM diagnosis and continued until the occurrence of the outcome of interest, death, or the end of the study period, as appropriate for each outcome. Participants contributed unexposed person-time before their first hospitalized TIA and exposed person-time thereafter. Thus, individuals who subsequently developed TIA were not classified as exposed from cohort entry. The models estimated adjusted hazard ratios (HRs) and 95% confidence intervals (CIs) for the association between time-varying TIA status and each study outcome.

All primary models were adjusted for prespecified baseline demographic, socioeconomic, lifestyle, medication, comorbidity, anthropometric, and laboratory characteristics. Covariates were selected based on clinical relevance and prior knowledge rather than on univariable statistical significance. For outcomes subject to competing mortality, cause-specific Cox models were fitted, with competing deaths treated as censoring events according to the prespecified outcome-specific follow-up definitions. The estimated HRs therefore represented relative differences in the instantaneous hazard among participants who remained at risk.

Exploratory subgroup analyses were performed according to sex, age (<60 vs ≥60 years), BMI (<24 vs ≥24 kg/m²), HbA1c (<7% vs ≥7%), and baseline insulin use. The BMI cutoff was based on the Chinese adult overweight classification (20). Effect modification was assessed by adding interaction terms between time-varying TIA status and each baseline characteristic. Age, BMI, and HbA1c were modeled as continuous variables in the interaction tests, whereas sex and baseline insulin use were modeled as categorical variables. Accordingly, the interaction P values for age, BMI, and HbA1c reflected effect modification across the continuous scale rather than a formal comparison between the displayed categories. Because these analyses were exploratory, no adjustment for multiple testing was applied.

A series of sensitivity analyses was conducted to examine the robustness of the primary findings. First, the transition from the unexposed to the exposed state was delayed by 7, 30, 90, and 180 days after the first hospitalized TIA. Under these definitions, person-time and events occurring before the delayed exposure date remained classified as unexposed, while cohort entry and the original outcome-specific follow-up were unchanged. Second, analyses were repeated among participants with documented smoking status and in models excluding ethnicity, marital status, and insurance type, which had relatively high proportions of missing data. Third, alternative TIA definitions were examined by restricting exposure to TIA recorded as the primary discharge diagnosis and by requiring at least two hospitalized TIA diagnoses. Ischemic stroke was additionally evaluated as a separate outcome.

Several supplementary descriptive analyses were also performed. Post-TIA event counts were summarized within cumulative 7-, 30-, 90-, and 180-day windows, using the corresponding outcome-specific post-TIA population as the denominator. In addition, age-specific incidence rates were calculated separately for TIA-unexposed and TIA-exposed person-time and expressed per 1,000 person-years. Absolute rate differences were calculated as the incidence rate during exposed person-time minus that during unexposed person-time within each age group, with corresponding 95% CIs. These incidence rates and rate differences were unadjusted descriptive measures and were reported separately from the adjusted HRs derived from the time-varying Cox models.

All analyses were performed using R version 4.4.1. Statistical tests were two-sided, with P<0.05 considered statistically significant for the primary analyses. Interaction P values from subgroup analyses were interpreted as exploratory.

## Results

### Baseline characteristics of the study population

Among 232,688 patients with newly diagnosed T2DM, 11,153 experienced a hospitalized TIA during follow-up and 221,535 did not. Participants who experienced TIA were older than those who did not (median age, 61 vs 55 years) and were more often female (55.73% vs 46.90%). They also had a higher prevalence of hypertension (56.61% vs 43.03%) and were more frequently covered by Urban Employee Basic Medical Insurance (53.48% vs 35.94%).

Differences were also observed in baseline medication use. Sulfonylureas, calcium channel blockers, ACE inhibitors, ARBs, and statins were more commonly used among participants who later experienced TIA, whereas metformin, GLP-1 receptor agonists, SGLT-2 inhibitors, and DPP-4 inhibitors were less commonly used. Median BMI and HbA1c were slightly lower in the TIA group (23.66 vs 24.03 kg/m² and 7.30% vs 7.50%, respectively). Other differences were observed across several comorbidities, medications, and laboratory measures (**Table 1**).

**Table 1:** Baseline characteristics by TIA incidence status during follow-up period.

Characteristic	Overall, N = 232,688	TIA during follow-up		*P* value
		No, N = 221,535	Yes, N = 11,153	
Female	110,123 (47.33%)	103,907 (46.90%)	6,216 (55.73%)	<0.001
Age (years)	56.00 (46.00, 65.00)	55.00 (46.00, 64.00)	61.00 (54.00, 69.00)	<0.001
Elderly	90,515 (38.90%)	84,261 (38.04%)	6,254 (56.07%)	<0.001
Ethnicity				<0.001
Han	152,202 (65.41%)	144,726 (65.33%)	7,476 (67.03%)	
Others	1,288 (0.55%)	1,245 (0.56%)	43 (0.39%)	
Unknown	79,198 (34.04%)	75,564 (34.11%)	3,634 (32.58%)	
Married				<0.001
Married	79,220 (34.05%)	75,879 (34.25%)	3,341 (29.96%)	
Unmarried/Others	16,902 (7.26%)	16,204 (7.31%)	698 (6.26%)	
Unknown	136,566 (58.69%)	129,452 (58.43%)	7,114 (63.79%)	
Insurance				<0.001
UEBMI	85,593 (36.78%)	79,628 (35.94%)	5,965 (53.48%)	
URBMI/Others	136,869 (58.82%)	131,858 (59.52%)	5,011 (44.93%)	
Unknown	10,226 (4.39%)	10,049 (4.54%)	177 (1.59%)	
Smoking history				<0.001
Yes	36,008 (15.47%)	34,459 (15.55%)	1,549 (13.89%)	
No	105,186 (45.20%)	98,982 (44.68%)	6,204 (55.63%)	
Unknown	91,494 (39.32%)	88,094 (39.77%)	3,400 (30.49%)	
Drinking history				<0.001
Yes	23,240 (9.99%)	22,378 (10.10%)	862 (7.73%)	
No	116,011 (49.86%)	109,246 (49.31%)	6,765 (60.66%)	
Unknown	93,437 (40.16%)	89,911 (40.59%)	3,526 (31.61%)	
BMI (kg/m^2^)	24.02 (21.71, 26.51)	24.03 (21.72, 26.56)	23.66 (21.47, 25.99)	<0.001
Other GLDs use				
Insulins	40,241 (17.29%)	38,645 (17.44%)	1,596 (14.31%)	<0.001
Metformin	39,628 (17.03%)	38,232 (17.26%)	1,396 (12.52%)	<0.001
Sulfonylureas	24,609 (10.58%)	22,821 (10.30%)	1,788 (16.03%)	<0.001
AGIs	19,664 (8.45%)	18,686 (8.43%)	978 (8.77%)	0.216
Thiazolidinediones	4,382 (1.88%)	4,229 (1.91%)	153 (1.37%)	<0.001
GLP-1 RAs	1,003 (0.43%)	998 (0.45%)	5 (0.04%)	<0.001
SGLT-2i	9,553 (4.11%)	9,483 (4.28%)	70 (0.63%)	<0.001
DPP-4i	10,265 (4.41%)	10,106 (4.56%)	159 (1.43%)	<0.001
Glinides	6,434 (2.77%)	5,993 (2.71%)	441 (3.95%)	<0.001
Other medication use				
Aspirin	27,876 (11.98%)	26,417 (11.92%)	1,459 (13.08%)	<0.001
Proton pump inhibitors	45,409 (19.51%)	43,641 (19.70%)	1,768 (15.85%)	<0.001
Diuretics	12,498 (5.37%)	11,945 (5.39%)	553 (4.96%)	0.047
β-Blocking agents	23,011 (9.89%)	21,704 (9.80%)	1,307 (11.72%)	<0.001
Calcium channel blockers	34,815 (14.96%)	32,222 (14.54%)	2,593 (23.25%)	<0.001
ACE inhibitors	9,701 (4.17%)	8,892 (4.01%)	809 (7.25%)	<0.001
ARBs	23,283 (10.01%)	21,916 (9.89%)	1,367 (12.26%)	<0.001
Statins	46,376 (19.93%)	43,952 (19.84%)	2,424 (21.73%)	<0.001
Other lipid-modifying agents	8,472 (3.64%)	8,176 (3.69%)	296 (2.65%)	<0.001
H2-receptor antagonists	2,509 (1.08%)	2,250 (1.02%)	259 (2.32%)	<0.001
Diabetes complications				
Diabetic retinopathy	5,111 (2.20%)	4,951 (2.23%)	160 (1.43%)	<0.001
Diabetic nephropathy	4,498 (1.93%)	4,357 (1.97%)	141 (1.26%)	<0.001
Diabetic neuropathy	4,937 (2.12%)	4,734 (2.14%)	203 (1.82%)	0.024
Diabetic foot	4,171 (1.79%)	4,061 (1.83%)	110 (0.99%)	<0.001
Other comorbidities				
Mood disorders	4,209 (1.81%)	3,939 (1.78%)	270 (2.42%)	<0.001
Anxiety	9,210 (3.96%)	8,606 (3.88%)	604 (5.42%)	<0.001
Psychosis	1,417 (0.61%)	1,379 (0.62%)	38 (0.34%)	<0.001
Angina pectoris	7,192 (3.09%)	6,751 (3.05%)	441 (3.95%)	<0.001
Atrial fibrillation	4,874 (2.09%)	4,714 (2.13%)	160 (1.43%)	<0.001
Hypertension	101,644 (43.68%)	95,330 (43.03%)	6,314 (56.61%)	<0.001
Venous thromboembolism	2,303 (0.99%)	2,251 (1.02%)	52 (0.47%)	<0.001
Peripheral vascular disease	45,951 (19.75%)	43,860 (19.80%)	2,091 (18.75%)	0.007
Chronic kidney disease	11,296 (4.85%)	10,943 (4.94%)	353 (3.17%)	<0.001
Hyperlipidemia	66,768 (28.69%)	63,291 (28.57%)	3,477 (31.18%)	<0.001
Liver disease	42,474 (18.25%)	41,116 (18.56%)	1,358 (12.18%)	<0.001
COPD	31,673 (13.61%)	29,740 (13.42%)	1,933 (17.33%)	<0.001
Asthma	5,654 (2.43%)	5,265 (2.38%)	389 (3.49%)	<0.001
Thyroid disease	16,671 (7.16%)	16,137 (7.28%)	534 (4.79%)	<0.001
Osteoporosis	19,559 (8.41%)	18,055 (8.15%)	1,504 (13.49%)	<0.001
Osteoarthritis	13,604 (5.85%)	12,494 (5.64%)	1,110 (9.95%)	<0.001
Rheumatoid arthritis	3,616 (1.55%)	3,277 (1.48%)	339 (3.04%)	<0.001
Gout	8,122 (3.49%)	7,762 (3.50%)	360 (3.23%)	0.121
Head injury	5,975 (2.57%)	5,730 (2.59%)	245 (2.20%)	0.011
Central nervous system infections	555 (0.24%)	537 (0.24%)	18 (0.16%)	0.087
Fracture	11,735 (5.04%)	11,229 (5.07%)	506 (4.54%)	0.012
Malignant neoplasms	18,181 (7.81%)	17,553 (7.92%)	628 (5.63%)	<0.001
Epilepsy	1,254 (0.54%)	1,170 (0.53%)	84 (0.75%)	0.002
Alzheimer's disease	1,196 (0.51%)	1,126 (0.51%)	70 (0.63%)	0.085
Laboratory test indicators				
HbA1c (%)	7.50 (6.50, 9.70)	7.50 (6.50, 9.70)	7.30 (6.40, 9.30)	<0.001
TC (mmol/L)	5.17 (4.33, 6.04)	5.16 (4.32, 6.03)	5.23 (4.41, 6.15)	<0.001
TG (mmol/L)	1.67 (1.14, 2.58)	1.67 (1.15, 2.60)	1.55 (1.08, 2.37)	<0.001
HDL (mmol/L)	1.22 (1.02, 1.46)	1.22 (1.02, 1.46)	1.27 (1.06, 1.52)	<0.001
LDL (mmol/L)	3.08 (2.89, 3.78)	3.08 (2.89, 3.78)	3.09 (2.39, 3.80)	0.081
Crea (µmol/L)	67.40 (55.80, 82.00)	67.40 (55.80, 82.00)	67.20 (56.00, 81.00)	0.407
UA (µmol/L)	324.00 (261.60, 395.00)	324.50 (262.00, 396.00)	316.00 (256.00, 384.80)	<0.001

ACE, angiotensin-converting enzyme; AGI, alpha-glucosidase inhibitor; ARB, angiotensin receptor blocker; BMI, body mass index; COPD, chronic obstructive pulmonary disease; Crea, creatinine; DPP-4i, dipeptidyl peptidase-4 inhibitor; GLD, glucose-lowering drug; GLP-1 RA, glucagon-like peptide-1 receptor agonist; HbA1c, glycated hemoglobin; HDL, high-density lipoprotein cholesterol; LDL, low-density lipoprotein cholesterol; SGLT-2i, sodium-glucose cotransporter-2 inhibitor; TC, total cholesterol; TG, triglycerides; TIA, transient ischemic attack; UA, uric acid; UEBMI, Urban Employee Basic Medical Insurance; URBMI, Urban and Rural Resident Basic Medical Insurance.

Data are presented as n (%) or median (interquartile range). P values were calculated using Pearson’s chi-square test or Wilcoxon rank-sum test, as appropriate. TIA status in this table is descriptive; TIA was modeled as a time-varying exposure in the primary analyses. Unknown indicates missing or undocumented information. Elderly was defined as age ≥60 years.

### Associations between time-varying TIA and clinical outcomes

In the time-varying Cox analyses, hospitalized TIA was associated with higher hazards of the cardiovascular composite, all-cause mortality, cardiovascular mortality, stroke, and heart failure. The adjusted HR was 2.07 (95% CI, 1.96–2.20) for the cardiovascular composite, 1.98 (95% CI, 1.87–2.10) for all-cause mortality, 2.31 (95% CI, 2.09–2.55) for cardiovascular mortality, 2.46 (95% CI, 2.32–2.61) for stroke, and 1.55 (95% CI, 1.48–1.63) for heart failure (all P<0.001). In contrast, the association with myocardial infarction was not statistically significant (HR, 1.10; 95% CI, 0.99–1.22; P = 0.071) (**Table 2**).

**Table 2:** HR of outcomes based on time-dependent TIA exposure.

Outcomes	Events / N	Time-varying Cox		
		Adjusted HR	95% CI	*P* value
Cardiovascular composite	62,440 / 232,688	2.07	1.96–2.20	<0.001
All-cause mortality	12,905 / 232,688	1.98	1.87–2.10	<0.001
Cardiovascular mortality	3,378 / 232,688	2.31	2.09–2.55	<0.001
Incident MI	9,816 / 232,688	1.1	0.99–1.22	0.071
Incident Stroke	42,786 / 232,688	2.46	2.32–2.61	<0.001
Incident HF	27,794 / 232,688	1.55	1.48–1.63	<0.001

CI, confidence interval; HF, heart failure; HR, hazard ratio; MI, myocardial infarction; TIA, transient ischemic attack.

Events denotes the total number of incident outcome events during follow-up, and N denotes the number of participants included in the corresponding analysis. The cardiovascular composite comprises myocardial infarction, stroke, heart failure, or cardiovascular mortality. All participants entered follow-up at T2DM diagnosis (the index date). TIA was modeled as a time-varying exposure. Participants contributed TIA-unexposed person-time before the first hospitalized TIA and TIA-exposed person-time thereafter. HRs were estimated using multivariable time-varying Cox proportional hazards models.

### Exploratory subgroup analyses

Exploratory subgroup analyses showed that the association between hospitalized TIA and the cardiovascular composite was consistently observed across prespecified subgroups (**Table 3**). The association was stronger among participants aged <60 years than among those aged ≥60 years (HR, 2.51 vs 1.83; P for interaction <0.001) and among those with baseline HbA1c ≥7% than among those with HbA1c <7% (HR, 2.27 vs 1.86; P for interaction <0.001). No significant interaction was observed for sex, BMI, or baseline insulin use.

**Table 3:** Subgroup analyses of the associations between time-varying hospitalized TIA and study outcomes.

Subgroup	N	Cardiovascular composite		All-cause mortality		Cardiovascular mortality		Incident MI		Incident Stroke		Incident HF	
		HR (95% CI)	*P*-Inter	HR (95% CI)	*P*-Inter	HR (95% CI)	*P*-Inter	HR (95% CI)	*P*-Inter	HR (95% CI)	*P*-Inter	HR (95% CI)	*P*-Inter
Sex													
Male	122,565	2.12 (1.95–2.31)	0.811	2.14 (1.99–2.31)	0.014	2.58 (2.25–2.95)	0.095	1.02 (0.89–1.17)	0.005	2.79 (2.56–3.05)	<0.001	1.55 (1.45–1.67)	0.347
Female	110,123	1.99 (1.85–2.15)		1.84 (1.69–2.01)		2.14 (1.85–2.48)		1.27 (1.08–1.50)		2.22 (2.06–2.41)		1.57 (1.47–1.69)	
Age group, years													
< 60	142,173	2.51 (2.30–2.74)	<0.001	2.02 (1.76–2.33)	0.025	2.41 (1.80–3.24)	0.036	1.06 (0.88–1.29)	0.915	2.96 (2.70–3.23)	<0.001	1.66 (1.51–1.83)	0.104
≥ 60	90,515	1.83 (1.70–1.97)		1.95 (1.84–2.07)		2.30 (2.07–2.56)		1.12 (0.98–1.27)		2.13 (1.97–2.30)		1.51 (1.43–1.61)	
Baseline BMI, kg/m^2^													
< 24	115,628	2.01 (1.86–2.17)	0.971	1.94 (1.81–2.08)	0.016	2.27 (2.01–2.58)	0.700	1.11 (0.97–1.29)	0.232	2.30 (2.12–2.49)	0.453	1.52 (1.42–1.63)	0.483
≥ 24	117,060	2.16 (1.99–2.35)		2.05 (1.86–2.25)		2.37 (2.02–2.79)		1.09 (0.93–1.28)		2.68 (2.46–2.91)		1.60 (1.48–1.72)	
Baseline HbA1c, %													
< 7	88,153	1.86 (1.70–2.03)	<0.001	1.90 (1.73–2.08)	<0.001	2.21 (1.89–2.60)	0.357	1.05 (0.89–1.24)	0.148	2.21 (2.02–2.43)	<0.001	1.53 (1.41–1.65)	0.003
≥ 7	144,535	2.27 (2.11–2.45)		2.04 (1.90–2.19)		2.39 (2.10–2.71)		1.15 (1.00–1.31)		2.67 (2.48–2.88)		1.57 (1.47–1.68)	
Insulins treatment													
No	192,447	2.06 (1.93–2.19)	0.481	1.90 (1.79–2.02)	0.003	2.29 (2.05–2.55)	0.427	1.06 (0.95–1.20)	0.255	2.43 (2.28–2.59)	0.639	1.51 (1.43–1.59)	0.011
Yes	40,241	1.99 (1.71–2.31)		2.21 (1.93–2.54)		2.24 (1.74–2.89)		1.20 (0.94–1.54)		2.40 (2.06–2.79)		1.65 (1.45–1.87)	

BMI, body mass index; CI, confidence interval; HbA1c, glycated hemoglobin; HF, heart failure; HR, hazard ratio; MI, myocardial infarction; TIA, transient ischemic attack; P-Inter, P values for interaction.

The cardiovascular composite comprises myocardial infarction, stroke, heart failure, or cardiovascular mortality. HRs were estimated using multivariable time-varying Cox proportional hazards models. *P* values for interaction were derived from multiplicative interaction terms between time-varying TIA exposure and the corresponding subgroup variable. Age, BMI, and HbA1c were continuous in interaction tests, although categorized for subgroup presentation; sex and insulin use were categorical. Interaction P values are nominal and exploratory, without multiplicity adjustment.

For stroke, the association was stronger in men than in women (HR, 2.79 vs 2.22; P for interaction <0.001), in participants aged <60 years than in those aged ≥60 years (HR, 2.96 vs 2.13; P for interaction <0.001), and in those with baseline HbA1c ≥7% than among those with HbA1c <7% (HR, 2.67 vs 2.21; P for interaction <0.001). No significant interaction was observed for BMI or baseline insulin use. Significant interactions were also observed for several other outcomes, including all-cause mortality, cardiovascular mortality, myocardial infarction, and heart failure.

### Sensitivity and supplementary analyses

The associations were generally robust across sensitivity analyses (**Table 4**). In the lagged analyses, the association between hospitalized TIA and the cardiovascular composite was attenuated as the lag period increased, with HRs of 1.97 (95% CI, 1.86–2.09), 1.85 (95% CI, 1.74–1.96), 1.76 (95% CI, 1.66–1.88), and 1.69 (95% CI, 1.58–1.80) for the 7-, 30-, 90-, and 180-day lags, respectively. A similar pattern was observed for stroke, with corresponding HRs of 2.32 (95% CI, 2.18–2.46), 2.15 (95% CI, 2.02–2.29), 2.04 (95% CI, 1.91–2.17), and 1.92 (95% CI, 1.79–2.06). In contrast, estimates for all-cause mortality remained close to 2.0 across lag periods (HRs, 1.98–1.99), as did those for cardiovascular mortality (HRs, 2.31–2.32), while the association with heart failure remained relatively stable (HRs, 1.52–1.55). No significant association with myocardial infarction was observed in any lagged analysis (HRs, 1.07–1.10).

**Table 4:** Sensitivity analyses of the associations between time-varying hospitalized TIA and study outcomes.

Sensitivity analyses	Cardiovascular composite	All-cause mortality	Cardiovascular mortality	Incident MI	Incident stroke	Incident ischemic stroke	Incident HF
	HR (95% CI)	HR (95% CI)	HR (95% CI)	HR (95% CI)	HR (95% CI)	HR (95% CI)	HR (95% CI)
Primary analysis							
Time-varying Cox model	2.07 (1.96–2.20)	1.98 (1.87–2.10)	2.31 (2.09–2.55)	1.10 (0.99–1.22)	2.46 (2.32–2.61)	2.45 (2.31–2.60)	1.55 (1.48–1.63)
Lagged analyses							
7-day lag	1.97 (1.86–2.09)	1.98 (1.87–2.09)	2.31 (2.09–2.55)	1.09 (0.98–1.22)	2.32 (2.18–2.46)	2.31 (2.18–2.46)	1.55 (1.47–1.63)
30-day lag	1.85 (1.74–1.96)	1.98 (1.87–2.09)	2.32 (2.10–2.56)	1.08 (0.97–1.21)	2.15 (2.02–2.29)	2.14 (2.01–2.28)	1.53 (1.45–1.61)
90-day lag	1.76 (1.66–1.88)	1.98 (1.87–2.10)	2.32 (2.10–2.57)	1.07 (0.96–1.20)	2.04 (1.91–2.17)	2.03 (1.90–2.17)	1.52 (1.44–1.60)
180-day lag	1.69 (1.58–1.80)	1.99 (1.87–2.10)	2.31 (2.08–2.55)	1.10 (0.98–1.23)	1.92 (1.79–2.06)	1.91 (1.78–2.05)	1.54 (1.46–1.62)
Missing-data analyses							
Complete-case analysis for smoking	1.86 (1.74–1.99)	1.87 (1.75–1.99)	2.30 (2.05–2.59)	1.02 (0.91–1.15)	2.25 (2.09–2.41)	2.21 (2.06–2.38)	1.41 (1.33–1.50)
Excluding covariates with substantial missingness	2.00 (1.89–2.12)	1.96 (1.85–2.07)	2.35 (2.13–2.60)	1.03 (0.93–1.15)	2.41 (2.27–2.56)	2.42 (2.28–2.57)	1.48 (1.41–1.55)
Alternative TIA definitions							
Primary discharge diagnosis only	2.39 (2.08–2.74)	1.97 (1.80–2.17)	2.33 (1.99–2.73)	0.99 (0.82–1.19)	3.43 (2.99–3.94)	3.13 (2.73–3.59)	1.35 (1.23–1.48)
≥2 hospitalized TIA diagnoses	3.14 (2.91–3.39)	1.79 (1.65–1.93)	1.92 (1.67–2.21)	1.11 (0.95–1.28)	3.86 (3.58–4.17)	3.88 (3.59–4.19)	1.71 (1.60–1.83)

CI, confidence interval; HF, heart failure; HR, hazard ratio; MI, myocardial infarction; TIA, transient ischemic attack.

The cardiovascular composite comprises myocardial infarction, stroke, heart failure, or cardiovascular mortality. The primary analysis modeled hospitalized TIA as a time-varying exposure. Lagged analyses delayed the exposure switch by 7, 30, 90, or 180 days; person-time and events before the delayed switch remained unexposed. Early events were retained, and follow-up was not restarted at a landmark. Missing-data analyses assessed alternative handling of incompletely recorded baseline covariates. Alternative TIA definitions examined primary discharge diagnosis and repeated hospitalized TIA records.

The timing of post-TIA events supported the use of these lagged analyses. Of all post-TIA cardiovascular composite events, 5.5%, 12.6%, 20.2%, and 28.5% occurred within 7, 30, 90, and 180 days, respectively; the corresponding proportions for stroke were 6.0%, 13.9%, 21.8%, and 30.5% (**Supplementary Table 2**).

Results were also similar in the missing-data analyses. The HR for the cardiovascular composite was 1.86 (95% CI, 1.74–1.99) in the complete-case analysis for smoking and 2.00 (95% CI, 1.89–2.12) after excluding ethnicity, marital status, and insurance type. Corresponding HRs for stroke were 2.25 (95% CI, 2.09–2.41) and 2.41 (95% CI, 2.27–2.56), respectively. Estimates for all-cause mortality, cardiovascular mortality, and heart failure were also broadly consistent with the primary analysis, whereas myocardial infarction remained null.

Associations were also observed under the alternative TIA definitions. When TIA was restricted to the primary discharge diagnosis, the HR was 2.39 (95% CI, 2.08–2.74) for the cardiovascular composite and 3.43 (95% CI, 2.99–3.94) for stroke. When at least two hospitalized TIA diagnoses were required, the corresponding HRs were 3.14 (95% CI, 2.91–3.39) and 3.86 (95% CI, 3.58–4.17). The associations with all-cause mortality, cardiovascular mortality, and heart failure remained elevated under both alternative definitions, whereas myocardial infarction remained unassociated with TIA.

Age-stratified analyses showed higher unadjusted incidence rates during TIA-exposed than TIA-unexposed person-time for the cardiovascular composite and stroke in both age groups (**Supplementary Table 3**). For the cardiovascular composite, the absolute rate difference was 46.73 per 1,000 person-years (95% CI, 40.44–53.03) among participants aged <60 years and 51.94 per 1,000 person-years (95% CI, 41.83–62.05) among those aged ≥60 years. For stroke, the corresponding differences were 46.83 (95% CI, 41.20–52.46) and 52.16 (95% CI, 44.19–60.13) per 1,000 person-years.

## Discussion

In this population-based cohort, hospitalized TIA was associated with higher subsequent hazards of the cardiovascular composite, stroke, HF, and mortality after T2DM diagnosis. The overall MI association did not reach statistical significance. By treating TIA as a time-varying exposure from cohort entry, the analysis distinguished pre-TIA and post-TIA person-time within a common follow-up framework. The study’s contribution is its diabetes-specific setting and broad outcome assessment, including HF and mortality. It does not establish that TIA causes the subsequent increase in cardiovascular risk.

The association with stroke is consistent with established evidence on cerebrovascular prognosis after TIA (7–11). TIAregistry.org documented substantial early stroke risk and persistent risk during longer follow-up (9, 10). Population-based studies similarly reported elevated stroke risk after TIA despite improvements in secondary prevention (21). A systematic review comparing inpatient and outpatient management also identified subsequent stroke as an important outcome across care settings (22). More recently, a Danish nationwide study found elevated long-term ischemic stroke incidence after first-time TIA (23). Our separate ischemic stroke analysis showed an association similar to that for the overall stroke endpoint.

Early post-TIA stroke requires particular care because diagnostic evolution, reclassification, or intensive investigation can link TIA and stroke records from the same clinical episode. In this cohort, 6.0% of post-TIA strokes occurred within 7 days and 30.5% within 180 days. Delaying the exposure switch progressively attenuated the stroke association, from an HR of 2.46 to 1.92 at 180 days. The cardiovascular composite showed a similar pattern. These analyses retained early events in the unexposed state rather than deleting them or selecting survivors at a landmark. They show that the association depends partly on the timing of exposure classification, while remaining elevated after a longer delay. They cannot determine whether an early stroke represents a separate event or evolution of the initial presentation.

The associations with the cardiovascular composite and mortality are consistent with the broader vascular burden documented after TIA. In the Dutch TIA Trial, 10-year risks were approximately 44% for major vascular events and 43% for mortality (24). Diabetes was associated with greater long-term mortality in that cohort (24). Other long-term observations documented both cerebrovascular and cardiac events after TIA or minor stroke (25). Our four-component composite explicitly included HF alongside MI, stroke, and cardiovascular mortality. This definition should be considered when comparing our estimates with studies using different cardiovascular composites.

MI differed from the other outcomes in the primary analysis. Previous studies documented clinically relevant MI risk after TIA or ischemic stroke, including an elevated rate relative to the general population (12, 26). Our comparison instead involved person-time within a T2DM cohort, in which underlying coronary risk was already substantial. The adjusted MI HR was 1.10, with a CI spanning unity. The findings therefore did not establish a statistically significant overall MI association, but they also did not establish equivalence between exposure states. Lower crude MI rates during exposed person-time and an adjusted HR slightly above unity represent different descriptive and adjusted comparisons. These findings do not support interpreting TIA as protective against MI.

The mortality associations reinforce the importance of considering outcomes beyond recurrent stroke. In the Dutch TIA Trial, diabetes was among the strongest predictors of long-term mortality (24). An Australian linkage study also found lower survival after TIA hospitalization than expected in an age- and sex-matched population (27). HF, atrial fibrillation, and previous stroke were important predictors of excess mortality in that study (27). Our results are consistent with TIA identifying patients with substantial vascular vulnerability and comorbidity, rather than an isolated neurological risk.

HF is a relevant addition to the assessment of cardiovascular prognosis after TIA in patients with diabetes. Previous studies linked cardiac sources of cerebral ischemia with adverse vascular outcomes and identified HF as a predictor of poor survival after TIA (25, 27, 28). The observed HF association may reflect shared cardiometabolic disease, including hypertension, atherosclerosis, atrial fibrillation, and diabetic myocardial dysfunction. Our analysis does not demonstrate a direct pathway from TIA to HF. It supports including HF in broader outcome assessment rather than restricting post-TIA prognosis to stroke alone.

Shared vascular pathology provides a plausible context for these associations. Diabetes is associated with endothelial dysfunction, inflammation, oxidative stress, and abnormalities of coagulation and fibrinolysis (4, 16–18). These processes affect multiple vascular beds and can contribute to both cerebral ischemia and cardiovascular disease. Registry evidence has also shown poorer outcomes after TIA or stroke among patients with diabetes (18). TIA can therefore mark accumulated vascular disease that was present before the hospitalization. The present observational data do not distinguish this explanation from changes in subsequent care or other evolving determinants of prognosis.

Age-stratified results illustrate the distinction between relative and absolute associations. Composite and stroke HRs were numerically larger among participants aged <60 years, consistent with age-related differences described in previous vascular studies (29, 30). However, older participants had higher exposed-period event rates and larger absolute rate differences for the composite, mortality, and HF. Thus, a larger relative HR in younger participants does not imply a greater absolute event burden. Interaction P values tested continuous age rather than the dichotomous age groups used for presentation. The unadjusted age-specific rates complement the adjusted HRs but do not provide adjusted causal risk differences.

The exploratory subgroup analyses indicated potential variation in the stroke association by sex, age, and HbA1c. Prior TIA studies reported sex differences in clinical profiles, neuroimaging findings, and long-term outcomes (31, 32). Such observations provide context but do not confirm the sex-related pattern in this cohort. The present analyses do not establish a sex-specific causal effect or support different management solely on the basis of these results.

Potential variation in the cardiovascular composite association was also observed across age and HbA1c. Previous research has linked abnormal glycemic status with adverse outcomes after ischemic stroke or TIA (33). Age and HbA1c were continuous in the interaction models, while categories were used to display subgroup estimates. The interaction tests therefore do not establish the displayed cutoffs as discrete risk or treatment boundaries. All subgroup analyses were exploratory and used nominal P values without multiplicity adjustment. These patterns are hypothesis-generating and require independent confirmation.

The clinical implication is that hospitalized TIA can identify patients with T2DM who warrant assessment of both cerebrovascular and broader cardiovascular risk. Established post-TIA evidence has emphasized subsequent stroke (7–11, 21–23), whereas this cohort also documented associations with HF and mortality. The findings neither establish the benefit of a specific post-TIA intervention nor quantify the incremental predictive value of adding TIA to a risk score. Prospective studies are needed to evaluate those questions.

The study’s strengths include its population-based design, large cohort, and linkage of diagnostic, prescription, laboratory, and mortality information. Excluding recorded prior TIA strengthened the definition of incident hospitalized TIA. The principal time-varying analysis allocated exposure according to its occurrence during follow-up, avoiding classification of pre-TIA person-time as exposed (34). Complementary analyses examined delayed exposure switching, missing-data handling, alternative TIA definitions, and ischemic stroke. Age-specific absolute rates added information that relative HRs alone could not provide.

### Limitations

Several limitations constrain interpretation. First, this observational study estimates prognostic associations rather than causal effects. Covariates were measured during the pre-index year and were not updated to represent health status immediately before TIA. Residual confounding can arise from changing blood pressure, glycemia, renal function, atrial fibrillation, frailty, medication use, and other unmeasured factors. Treatment changes and intensified surveillance after TIA can also influence subsequent outcomes. The findings therefore describe prognostic associations rather than causal effects of TIA. Second, T2DM entry was based on the first recorded diagnosis. Historical diagnosis and prescription records included pre-2010 information, but a uniform observation window was not available. Diabetes predating the available records or diagnosed outside the database could therefore remain unrecognized. Likewise, exclusion of recorded prior TIA did not establish lifetime absence of TIA. Third, code-based ascertainment can misclassify TIA and clinical outcomes. Neurological findings, imaging, symptom duration, and TIA mechanisms were incompletely captured. Hospitalized TIA represents a selected subset of all TIA, limiting applicability to outpatient events. Early stroke can also represent diagnostic evolution of the same presentation; delayed exposure-switch analyses do not resolve individual event classification. Alternative TIA definitions supported consistency but could not resolve these sources of bias. Finally, exclusion of advanced kidney disease and the setting of a single Chinese city constrain generalizability. Studies in additional populations and healthcare systems are needed to assess reproducibility.

## Conclusions

Among adults with newly diagnosed T2DM, incident hospitalized TIA was associated with higher subsequent hazards of the cardiovascular composite, all-cause mortality, cardiovascular mortality, HF, and stroke. The overall MI association did not reach statistical significance. These findings describe a broader adverse prognosis after hospitalized TIA and support assessing individual cardiovascular outcomes alongside the composite. The associations do not establish causality, and exploratory subgroup patterns require confirmation. Further prospective studies should clarify the contributions of evolving disease burden and post-TIA care to these outcomes.

## Data Availability

The data that support the findings of this study are available from the corresponding author upon reasonable request.

## Supplementary material

Supplementary Table 1 Missingness and handling of baseline covariates.

Supplementary Table 2 Timing of study outcomes after hospitalized transient ischemic attack.

Supplementary Table 3 Age-stratified incidence rates and absolute rate differences for study outcomes according to time-varying TIA exposure status.
