# Supplementary material for: Incident transient ischemic attack and subsequent cardiovascular events and mortality in patients with newly diagnosed type 2 diabetes: a population-based cohort study
Source: Front Endocrinol (Lausanne). 2026 Oct 28;17:1938124. doi: 10.3389/fendo.2026.1938124 (PMC13752093; doi:10.3389/fendo.2026.1938124)
Supplement: Supplementary file 1 [file Table1.docx]

| **Supplementary Table 1. Missingness and handling of baseline covariates** | | |
| --- | --- | --- |
| **Baseline covariate** | **Missing, N (%)** | **Handling in primary analysis** |
| Sex | 0 (0.00%) | No missing data |
| Age | 0 (0.00%) | No missing data |
| Ethnicity | 79,198 (34.04%) | Missing/unknown category |
| Marital status | 136,566 (58.69%) | Missing/unknown category |
| Insurance status | 10,226 (4.39%) | Missing/unknown category |
| Smoking history | 91,494 (39.32%) | Missing/unknown category |
| Drinking history | 93,437 (40.16%) | Missing/unknown category |
| BMI | 41,884 (18.00%) | Multiple imputation |
| HbA1c (%) | 55,845 (24.00%) | Multiple imputation |
| TC (mmol/L) | 39,557 (17.00%) | Multiple imputation |
| TG (mmol/L) | 34,903 (15.00%) | Multiple imputation |
| HDL (mmol/L) | 25,596 (11.00%) | Multiple imputation |
| LDL (mmol/L) | 44,211 (19.00%) | Multiple imputation |
| Crea (µmol/L) | 65,153 (28.00%) | Multiple imputation |
| UA (µmol/L) | 30,249 (13.00%) | Multiple imputation |
| **Abbreviations:** BMI, body mass index; Crea, creatinine; HbA1c, glycated hemoglobin; HDL, high-density lipoprotein cholesterol; LDL, low-density lipoprotein cholesterol; TC, total cholesterol; TG, triglycerides; UA, uric acid. | | |
|  |  |  |
| **Note:** Missingness was assessed before imputation, with percentages calculated using the total analytic cohort (N = 232,688) as the denominator. Missing categorical covariates were retained as a separate unknown category, whereas missing BMI and laboratory variables were handled using multiple imputation by chained equations. | | |
|  |  |  |
|  |  |  |

| **Supplementary Table 2. Timing of study outcomes after hospitalized transient ischemic attack** | | | | | |
| --- | --- | --- | --- | --- | --- |
| **Outcome** | **Total post-TIA events, N** | **≤7 days, N (%)** | **≤30 days, N (%)** | **≤90 days, N (%)** | **≤180 days, N (%)** |
| Cardiovascular composite | 1,251 | 69 (5.5%) | 158 (12.6%) | 253 (20.2%) | 356 (28.5%) |
| All-cause mortality | 1,460 | 6 (0.4%) | 16 (1.1%) | 48 (3.3%) | 91 (6.2%) |
| Cardiovascular mortality | 476 | 1 (0.2%) | 4 (0.8%) | 13 (2.7%) | 31 (6.5%) |
| Incident MI | 370 | 4 (1.1%) | 12 (3.2%) | 28 (7.6%) | 38 (10.3%) |
| Incident Stroke | 1,169 | 70 (6.0%) | 163 (13.9%) | 255 (21.8%) | 357 (30.5%) |
| Incident ischemic stroke | 1,144 | 67 (5.9%) | 160 (14.0%) | 250 (21.9%) | 350 (30.6%) |
| Incident HF | 1,679 | 11 (0.7%) | 50 (3.0%) | 113 (6.7%) | 176 (10.5%) |
| **Abbreviations:** HF, heart failure; MI, myocardial infarction; TIA, transient ischemic attack. | | | | | |
| **Note:** Post-TIA events were defined separately for each outcome using outcome-specific follow-up. The cardiovascular composite comprises myocardial infarction, stroke, heart failure, or cardiovascular mortality. Total post-TIA event counts are not directly comparable across outcomes. For example, a participant with a composite component event before TIA and heart failure after TIA would contribute to the post-TIA heart failure count, but not the post-TIA composite count. The 7-, 30-, 90-, and 180-day windows are cumulative. Percentages use the total post-TIA events for the corresponding outcome as the denominator. Events recorded on the same calendar date as TIA were not classified as post-TIA events. | | | | | |
|  |  |  |  |  |  |
|  |  |  |  |  |  |
|  |  |  |  |  |  |

| **Supplementary Table 3. Age-stratified incidence rates and absolute rate differences for study outcomes according to time-varying TIA exposure status** | | | | | | |
| --- | --- | --- | --- | --- | --- | --- |
| **Outcome** | **Age group (years)** | **TIA-unexposed** | | **TIA-exposed** | | **Absolute rate difference (95% CI)** |
|  |  | **Events / PY** | **Incidence rate** | **Events / PY** | **Incidence rate** |  |
| Cardiovascular composite | < 60 | 24,797 / 911,783 | 27.2 | 532 / 7,196 | 73.93 | 46.73 (40.44 - 53.03) |
|  | ≥ 60 | 36,392 / 424,086 | 85.81 | 719 / 5,219 | 137.75 | 51.94 (41.83 - 62.05) |
| All-cause mortality | < 60 | 2,908 / 1,023,882 | 2.84 | 222 / 33,014 | 6.72 | 3.88 (2.99 - 4.77) |
|  | ≥ 60 | 8,537 / 585,205 | 14.59 | 1,238 / 37,593 | 32.93 | 18.34 (16.48 - 20.20) |
| Cardiovascular mortality | < 60 | 527 / 1,023,882 | 0.51 | 52 / 33,014 | 1.58 | 1.06 (0.63 - 1.49) |
|  | ≥ 60 | 2,375 / 585,205 | 4.06 | 424 / 37,593 | 11.28 | 7.22 (6.13 - 8.31) |
| Myocardial infarction | < 60 | 4,241 / 1,004,953 | 4.22 | 111 / 31,529 | 3.52 | −0.70 (−1.37 - −0.03) |
|  | ≥ 60 | 5,205 / 562,084 | 9.26 | 259 / 35,723 | 7.25 | −2.01 (−2.93 - −1.09) |
| Stroke | < 60 | 16,532 / 950,044 | 17.4 | 501 / 7,799 | 64.23 | 46.83 (41.20 - 52.46) |
|  | ≥ 60 | 25,085 / 477,016 | 52.59 | 668 / 6,377 | 104.75 | 52.16 (44.19 - 60.13) |
| Ischemic stroke | < 60 | 15,315 / 955,764 | 16.02 | 491 / 8,003 | 61.35 | 45.33 (39.90 - 50.76) |
|  | ≥ 60 | 23,839 / 482,070 | 49.45 | 653 / 6,703 | 97.42 | 47.97 (40.47 - 55.47) |
| Heart failure | < 60 | 9,522 / 984,043 | 9.68 | 459 / 29,481 | 15.57 | 5.89 (4.46 - 7.33) |
|  | ≥ 60 | 16,593 / 516,202 | 32.14 | 1,220 / 29,186 | 41.8 | 9.66 (7.26 - 12.05) |
| **Abbreviations:** CI, confidence interval; HF, heart failure; MI, myocardial infarction; PY, person-years; TIA, transient ischemic attack. | | | | | | |
|  |  |  |  |  |  |  |
| **Notes:** The cardiovascular composite comprises myocardial infarction, stroke, heart failure, or cardiovascular mortality. Incidence rates and absolute rate differences are unadjusted and expressed per 1,000 person-years. Age was defined at T2DM diagnosis (the index date). TIA was a time-varying exposure, and person-time ended at outcome-specific follow-up termination. Participants contributed unexposed person-time before the first hospitalized TIA and exposed person-time thereafter, if still at risk for the corresponding outcome. Same-day TIA and outcome records remained unexposed. Participants without TIA remained unexposed throughout follow-up. Absolute rate differences were calculated as exposed minus unexposed incidence rates within each age group. | | | | | | |
|  |  |  |  |  |  |  |
|  |  |  |  |  |  |  |
|  |  |  |  |  |  |  |
|  |  |  |  |  |  |  |
